# Serum copper-to-zinc ratio and risk of incident pneumonia in caucasian men: a prospective cohort study

**DOI:** 10.1007/s10534-022-00414-4

**Published:** 2022-07-04

**Authors:** Setor K. Kunutsor, Ari Voutilainen, Jari A. Laukkanen

**Affiliations:** 1 National Institute for Health Research Bristol Biomedical Research Centre, University Hospitals Bristol and Weston NHS Foundation Trust and the University of Bristol, Bristol, UK; 2 Musculoskeletal Research Unit, Translational Health Sciences, Bristol Medical School, University of Bristol, Learning & Research Building (Level 1), Southmead Hospital, Bristol, BS10 5NB UK; 3 Department of Medicine, Central Finland Health Care District Hospital District, Jyvaskyla, Finland; 4 Diabetes Research Centre, University of Leicester, Leicester General Hospital, Gwendolen Road, Leicester, LE5 4WP UK; 5 Institute of Public Health and Clinical Nutrition, University of Eastern Finland, Kuopio, Finland; 6 Institute of Clinical Medicine, Department of Medicine, University of Eastern Finland, Kuopio, Finland

**Keywords:** Serum copper-to-zinc ratio, Serum copper, Serum zinc, Pneumonia, Risk factor, Cohort study

## Abstract

Serum copper (Cu) and zinc (Zn), essential micronutrients that have important immunomodulatory and antimicrobial properties, are biomarkers of ageing. Serum Cu/Zn-ratio may be a more reliable marker for age-related degenerative conditions compared with serum Cu or Zn alone. We aimed to assess the association between Cu/Zn-ratio and the risk of incident pneumonia in a prospective cohort study. Serum levels of Cu and Zn were measured at baseline using atomic absorption spectrometry in 2503 men aged 42–61 years in the Kuopio Ischemic Heart Disease prospective cohort study. Hazard ratios (HRs) with confidence intervals (CIs) were calculated for incident pneumonia using Cox regression models. A total of 599 cases of pneumonia occurred during a median follow-up of 26.1 years. Serum Cu/Zn-ratio and Cu were each linearly associated with incident pneumonia. A unit increase in Cu/Zn-ratio was associated with an increased risk of pneumonia in analysis adjusted for potential confounders including C-reactive protein (HR 1.65; 95% CI 1.17–2.33). The corresponding adjusted HR (95% CI) was 2.04 (1.22–3.40) for serum Cu. The association between serum Zn and pneumonia was curvilinear. Compared to the bottom tertile of Zn, the multivariable adjusted HRs (95% CIs) for incident pneumonia were 0.68 (0.55–0.83) and 0.96 (0.79–1.16) for the middle and top tertiles of Zn, respectively. Further analysis in the same participants showed that Cu/Zn-ratio might be a stronger risk indicator for pneumonia than serum C-reactive protein. In middle-aged and older Finnish men, increased serum Cu/Zn-ratio and Cu concentrations are each linearly associated with an increased risk of incident pneumonia.

**Supplementary Information:**

The online version contains supplementary material available at 10.1007/s10534-022-00414-4.

## Introduction

Pneumonia, an inflammatory condition of the lung tissue commonly caused by bacteria or viruses, can be acquired in the community (community acquired pneumonia, CAP) or in the hospital environment (hospital acquired pneumonia). (Cilloniz et al. 2016b) Community-acquired pneumonia is a leading cause of hospitalization, morbidity, mortality, and associated with significant health care costs. (Nair and Niederman 2011)The Global Burden of Disease Study 2019 reported that lower respiratory infections ranked as the fourth leading cause of disability-adjusted life-years. (GBD Collaborators 2020) Despite the development of newer molecular tests for microbial identification of pathogens, pulmonary imaging facilities and antimicrobial therapies for the management of pneumonia over the last decade, the incidence of pneumonia persistently remains high. (Cillóniz et al. 2018) Major contributors to the growing incidence of pneumonia include increased life expectancy, smoking, excessive alcohol consumption, respiratory conditions such as asthma and chronic obstructive pulmonary disease (COPD), other chronic conditions such as diabetes, kidney and liver disease, and immunosuppression. Cillóniz and others 2018; Nair and Niederman 2011).

Though pneumonia constitutes a substantial global public health burden, it is a preventable cause of death and disability. Older age is a major risk factor for pneumonia, (Torres et al. 2013) which is a leading cause for hospitalization and subsequent mortality among this population group. (Jackson et al. 2004) This is due to the physiological changes associated with aging such as age-related weakening of the immune system (immunosenescence)(Haase and Rink 2009) as well as the high prevalence of chronic disease in older people. (Cilloniz et al. 2016a) With increasing life expectancy, there is increasing research focussed on identifying biomarkers of ageing,(Engelfriet et al. 2013) which could be clinically relevant for preventing aging-related diseases such as pneumonia. Copper (Cu) and zinc (Zn), essential micronutrients involved in several cellular processes such as nucleic acid synthesis, enzymatic reactions, oxidoreductases, inflammation, mitochondrial electron transport, cell replication and repair,(Chimienti 2013; Festa and Thiele 2011) have been identified as biomarkers related to aging as they appear to be mostly related to inflammatory parameters than the nutritional ones. (Malavolta et al.^2010^) They have important immunomodulatory and antimicrobial properties(Malavolta et al. 2015) and are relevant for the development, regulation and maintenance of the immune and antioxidative defence system. (Stafford et al. 2013) Copper is involved in various biological processes, and its insufficiency, deficiency, or toxic levels can lead to many disease states. (DiNicolantonio et al. 2018) Zinc deficiency contributes to frailty, disability and an increased incidence of age-related degenerative diseases such as cancer, infections and atherosclerosis. (Mocchegiani 2007).

Among serum micronutrients, concentrations of Cu and Zn are strictly regulated by compensatory mechanisms that act to stabilize them within certain ranges of nutritional intake.(Malavolta et al. 2015) Serum concentrations of Cu and Zn are only slightly affected by nutritional changes unless during severe deficiency or supplementation use.(Malavolta et al. 2015) However, in the presence of pathological changes such as inflammatory conditions, there is a decrease in serum Zn concentrations and an increase in serum Cu concentrations, and thus they are biologically interrelated.(Sullivan et al. 1979) The typical presentation of several age-related chronic diseases is an increase in the Cu-to-Zn ratio (Cu/Zn-ratio).(Malavolta et al. 2015) It has been suggested that the serum Cu/Zn-ratio may be a more reliable marker of pathological outcomes, compared to the use of Cu or Zn alone.(Malavolta et al. 2015) High serum Cu/Zn-ratio has been shown to be associated with an increased risk of cardiovascular mortality, (Leone et al. 2006) cancer, (Leone et al. 2006) all-cause mortality(Malavolta et al. 2010) as well as infectious disease. (Laine et al. 2020) Though the previous study by Laine and colleagues evaluated infection outcomes, the specific outcome of pneumonia was not assessed. (Laine et al. 2020) To our knowledge, the prospective association between serum Cu/Zn-ratio and the risk of the specific outcome of pneumonia has not been previously explored. Our principal aim was to assess the nature and magnitude of the prospective association of serum Cu/Zn-ratio with pneumonia risk, using a population-based prospective cohort of 2503 middle-aged and older Finnish men. A secondary aim was to assess the individual associations of serum Cu and Zn with incident pneumonia risk. Furthermore, given that C-reactive protein (CRP) is a major inflammatory marker, we also evaluated the association of serum high sensitivity CRP (hsCRP) with pneumonia risk in the same set of participants to make comparisons.

## Methods

This study was conducted in accordance with STROBE (STrengthening the Reporting of OBservational studies in Epidemiology) guidelines for reporting observational studies in epidemiology (Supplementary File 1).

### Study design and participants

The Research Ethics Committee of the University of Kuopio approved the study protocol and each study participant provided written informed consent. All study procedures adhered to the Declaration of Helsinki. Participants included in this study were part of the Kuopio Ischaemic Heart Disease Risk Factor Study (KIHD), a population-based prospective cohort study that was set up to investigate risk factors for atherosclerotic cardiovascular disease and other related diseases. The study design and recruitment methods have been described in detail in previous reports. (Kunutsor et al. 2016b, c; Laukkanen et al. 2018) Briefly, participants included in the KIHD comprised a representative sample of men aged 42, 48, 54 or 60 years living in the city of Kuopio and its surrounding rural communities in eastern Finland. During recruitment, a total of 3433 men were potentially eligible and of these, 3235 were found to be eligible for inclusion into study. Of this number, 2682 volunteered to participate and 553 did not respond to the invitation or declined to give informed consent. Baseline examinations were performed between March 1984 and December 1989. From this analysis, we excluded those with missing data on the exposures and potential confounders (n = 179). The current analysis included 2503 men with complete information on serum measurements of Cu and Zn, relevant covariates, and incident pneumonia events.

### Measurement of covariates and outcome ascertainment

Blood sample collection and measurement of blood biomarkers, physical measurements, and assessment of lifestyle characteristics, medical history and dietary intakes have been described in detail in previous reports. (Abdollahi et al. 2019; Kunutsor and others 2016a; Kunutsor and Laukkanen 2016; Salonen et al. 1992) Participants fasted overnight and abstained from drinking alcohol for at least 3 days and from smoking for at least 12 h before blood samples were taken between 8 and 10 a.m. Serum hsCRP measurements were made with an immunometric assay (Immulite High Sensitivity C-Reactive Protein Assay; DPC, Los Angeles, CA, USA). Measurements of serum Cu and Zn concentrations were made from frozen serum samples stored at −20° C for 1–5 years, using the PerkinElmer 306 atomic absorption spectrophotometer (Norwalk, Connecticut, USA). Self-administered questionnaires were used to assess medical history and lifestyle characteristics such as smoking and alcohol consumption. (Salonen et al. 1992) Socioeconomic status (SES) was assessed using self-reported questionnaires via a summary index that combined income, education, occupational prestige, material standard of living and housing conditions. The composite SES index ranged from 0 to 25, with higher values indicating lower SES. (Jae et al. 2020) The consumption of foods was assessed with the use of a 4-day guided food record, during three weekdays and one weekend day using household measures. Instructions were provided and completed food records were checked by a nutritionist together with the participant, to ensure accuracy. Leisure-time physical activity was assessed from a 12 month physical activity history modified from the Minnesota Leisure-Time Physical Activity Questionnaire. (Taylor et al. 1978).

Incident cases of pneumonia that occurred from study entry to 2018 were included in this analysis. The diagnoses of pneumonia cases were made by qualified physicians based on the International Classification of Diseases (ICD) codes used in clinical practice (ICD-8 codes 485; ICD-9 codes 480–483, and 485; ICD-10 codes J15, and J18) and were collected by linkage to the National Hospital Discharge Register (THL/93/5.05.00/2013). (Kunutsor et al. 2016a; Kunutsor et al. 2016b).

### Statistical analysis

Variables with skewed distributions (e.g., alcohol consumption, physical activity, and hsCRP) were natural log transformed to achieve approximately symmetrical distributions. Baseline characteristics were presented as means ± standard deviation (SD) or median (interquartile range, IQR) for continuous variables and n (percentages) for categorical variables. In linear regression models adjusted for age, Pearson correlation coefficients were calculated to assess the cross-sectional associations of serum Cu/Zn-ratio with various continuous risk markers; for categorical variables, the percentage differences in mean values of serum Cu/Zn-ratio for a category versus its reference were calculated. Hazard ratios (HRs) with 95% confidence intervals (CIs) for incident pneumonia were estimated using Cox proportional hazard models and these were adjusted for in three models: (Model 1) age; (Model 2) Model 1 plus body mass index (BMI), smoking status, history of type 2 diabetes, prevalent coronary heart disease (CHD), history of asthma, history of chronic bronchitis, history of tuberculosis, alcohol consumption, SES, leisure-time physical activity, total energy intake, intake of fruits, berries and vegetables, and intake of processed and unprocessed red meat; and (Model 3) Model 2 plus hsCRP (a potential mediator of the association). The selected confounders were based on their previously established roles as risk factors for pneumonia, evidence from previous research, or their potential as confounders based on known associations with incident pneumonia and observed associations with the exposures using the available data. (Groenwold et al. 2011) To explore potential nonlinear dose–response relationships between the exposures and incident pneumonia risk, we constructed multivariable restricted cubic splines (RCSs) with knots at the 5th, 35th, 65th, and 95th percentiles of the distribution of the exposures as recommended by Harrell. (Harrell 2001) Serum Cu/Zn-ratio and Cu were modeled as both continuous (per unit increase) and categorical (tertiles) variables given evidence of linear relationships with pneumonia risk using multivariable RCS curves; serum Zn was modeled as tertiles given evidence of a nonlinear relationship. We constructed Kaplan–Meier curves for tertiles of serum Cu/Zn-ratio and compared them using the log rank test. We used formal tests of interaction tests to assess statistical evidence of effect modification by clinically relevant characteristics. To minimize any bias due to reverse causation, sensitivity analysis involved excluding the first two years of follow-up. All statistical analyses were conducted using Stata version MP 17 (Stata Corp, College Station, Texas).

## Results

### Baseline characteristics

The overall mean (SD) age of study participants at recruitment was 53 (5) years. The means (SDs) of serum Cu/Zn-ratio, Cu and Zn were 1.21 (0.27), 1.11 (0.18) and 0.94 (0.12), respectively. Significant weak and positive correlations were observed between serum Cu/Zn-ratio and age, alcohol consumption, and SES; whereas, the correlation was stronger for hsCRP (r = 0.42). Significant weak and inverse correlations were observed with physical activity and intake of fruits, berries and vegetables. Values of serum Cu/Zn-ratio were significantly higher in men who smoked compared with men who did not smoke (Table 1).

**Table 1:** Baseline characteristics of study participants and cross-sectional correlates of copper-to-zinc ratio

Characteristics	Mean ± SD or median (IQR)	Pearson correlation r (95% CI)^a^	Percentage difference (95% CI) in values of percentage of Cu/Zn-ratio per 1 SD higher or compared to reference category of correlate^b^
Serum copper-to-zinc ratio	1.21 ± 0.27	–	–
Serum copper, mg/l	1.11 ± 0.18	–	–
Serum zinc, mg/l	0.94 ± 0.12	–	–
Questionnaire/prevalent conditions			
Age (years)	53 ± 5	0.11 (0.07, 0.15)***	0.03% (0.02, 0.04)***
Alcohol consumption, g/week	31.8 (6.2–91.0)	0.16 (0.12, 0.20)***	0.04% (0.03, 0.06)***
History of type 2 diabetes, n (%)			
No	2404 (96.0)	–	Ref
Yes	99 (4.0)	–	0.01% (−0.05, 0.06)
Current smoking, n (%)			
No	1712 (68.4)	–	Ref
Yes	791 (31.6)	–	0.11% (0.09, 0.14)***
History of CHD, n (%)		–	
No	1886 (75.4)	–	Ref
Yes	617 (24.6)	–	0.03% (0.00, 0.05)*
History of asthma, n (%)			
No	2412 (96.4)	–	Ref
Yes	91 (3.6)	–	0.02% (−0.04, 0.08)
History of chronic bronchitis, n (%)			
No	2314 (92.4)	–	Ref
Yes	189 (7.6)	–	0.01% (−0.03, 0.05)
History of tuberculosis, n (%)			
No	2406 (96.1)	–	Ref
Yes	97 (3.9)	–	−0.01% (−0.07, 0.04)
Physical measurements			
BMI, kg/m^2^	26.9 ± 3.6	−0.03 (−0.06, 0.01)	−0.01% (−0.02, 0.00)
SBP, mmHg	134 ± 17	0.02 (−0.02, 0.06)	0.01% (−0.00, 0.02)
DBP, mmHg	89 ± 11	−0.01 (−0.05, 0.03)	−0.00 (−0.01, 0.01)
Physical activity, KJ/day	1204 (630–1999)	−0.04 (−0.08, −0.00)*	−0.01% (−0.02, −0.00)*
Socio-economic status	8.48 ± 4.23	0.13 (0.09, 0.17)***	0.04% (0.02, 0.05)***
Blood-based markers			
Total cholesterol, mmol/l	5.90 ± 1.08	0.03 (−0.01, 0.06)	0.01% (−0.00, 0.02)
HDL-C, mmol/l	1.29 ± 0.30	0.01 (−0.03, 0.05)	0.00% (−0.01, 0.01)
Fasting plasma glucose, mmol/l	5.35 ± 1.28	0.03 (−0.00, 0.07)	0.01 (−0.00, 0.02)
High sensitivity C-reactive protein, mg/l	1.29 (0.71–2.48)	0.42 (0.38, 0.45)***	0.11% (0.10, 0.12)***
Dietary intakes			
Total energy intake, kJ/day	9855 ± 2595	0.00 (−0.04, 0.04)	0.00% (−0.01, 0.01)
Processed and unprocessed red meat, g/day	145 ± 77	0.04 (−0.00, 0.08)	0.01% (−0.00, 0.02)
Fruits, berries and vegetables, g/day	251 ± 156	−0.12 (−0.16, −0.08)***	−0.03 (−0.04, −0.02)***

BMI, body mass index; CHD, coronary heart disease; DBP, diastolic blood pressure; HDL-C, high-density lipoprotein cholesterol; SD, standard deviation; SBP, systolic blood pressure

^a^Pearson correlation coefficients between serum Cu/Zn-ratio and the row variables

^b^Percentage change in values of serum Cu/Zn-ratio per 1-SD increase in the row variable (or for categorical variables, the percentage difference in mean values of serum Cu/Zn-ratio for the category versus the reference); asterisks indicate the level of statistical significance: *, p < 0.05; **, p < 0.01; ***, p < 0.001

### Association of serum Cu/Zn-ratio with pneumonia

**Fig. 1:**
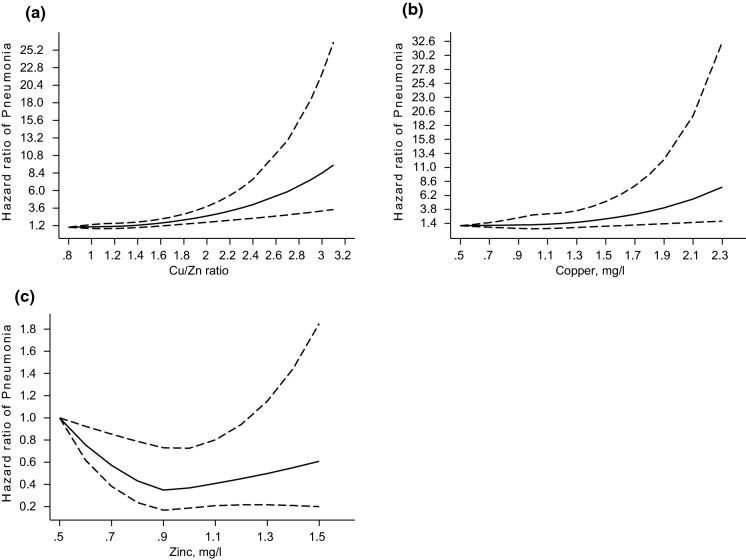
Restricted cubic splines of the hazard ratios of incident pneumonia with serum Cu/Zn-ratio, Cu and Zn **A** Serum Cu/Zn-ratio and pneumonia; **B** Serum Cu and pneumonia; **C** Serum Zn and pneumonia Dashed lines represent the 95% confidence intervals for the spline model (solid line). Models were adjusted for age, body mass index, smoking status, history of type 2 diabetes, prevalent coronary heart disease, history of asthma, history of chronic bronchitis, history of tuberculosis, alcohol consumption, socioeconomic status, leisure-time physical activity, total energy intake, intake of fruits, berries and vegetables, and intake of processed and unprocessed red meat Cu, copper; Zn, zinc

**Fig. 2:**
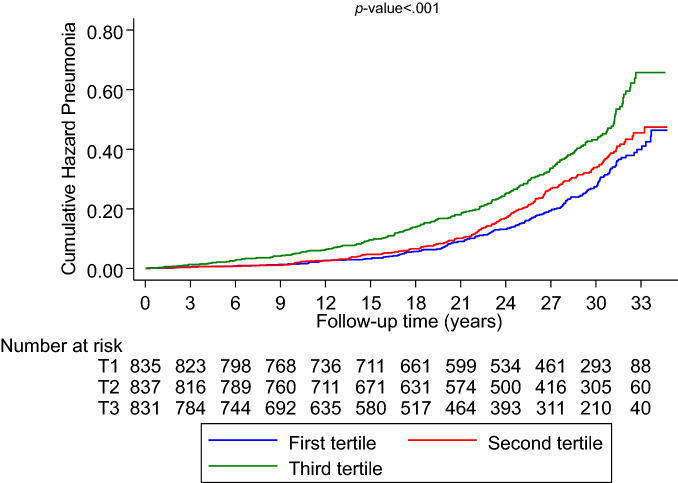
Cumulative Kaplan–Meier curves for pneumonia during follow-up according to tertiles of serum Cu/Zn-ratio

A total of 599 incident cases of pneumonia were recorded (annual rate 10.33/1000 person-years at risk; 95% CI 9.53–11.19) during a median (IQR) follow-up of 26.1 (16.7–30.8) years. A multivariable RCS curve showed that the risk of pneumonia increased linearly with increasing serum Cu/Zn-ratio across the range 1.50–3.10 (*p*-value for nonlinearity = 0.16) (Fig. 1A). The HR (95% CI) for incident pneumonia per unit increase in serum Cu/Zn-ratio was 2.07 (1.51–2.84) in analysis adjusted for age, BMI, smoking status, history of type 2 diabetes, prevalent CHD, history of asthma, chronic bronchitis or tuberculosis, alcohol consumption, SES, leisure-time physical activity, total energy intake, intake of fruits, berries and vegetables, and intake of processed and unprocessed red meat, which was attenuated to 1.65 (1.17–2.33) after further adjustment for hsCRP (Table 2). The corresponding adjusted HRs (95% CIs) were 1.32 (1.08–1.62) and 1.15 (0.92–1.42) comparing the top versus bottom tertiles of serum Cu/Zn-ratio. Cumulative hazard curves showed an increased risk of pneumonia among men in the top tertile of serum Cu/Zn-ratio compared with the other Cu/Zn-ratio groups (*p*-value for log-rank test < 0.001; Fig. 2).

**Table 2:** Associations of serum copper, zinc and copper-to-zinc ratio with risk of pneumonia

Exposure	Events/total	Model 1	*P-*value	Model 2	*P-*value	Model 3	*P-*value
		HR (95% CI)		HR (95% CI)		HR (95% CI)	
Serum copper-to-zinc ratio							
Per unit increase	599/2503	2.59 (1.92–3.49)	< 0.001	2.07 (1.51–2.84)	< .001	1.65 (1.17–2.33)	.004
T1 (0.48–1.07)	180/835	Ref		Ref		Ref	
T2 (1.08–1.27)	198/837	1.13 (0.92–1.38)	.25	1.07 (0.87–1.31)	.53	1.01 (0.82–1.24)	.92
T3 (1.28–3.12)	221/831	1.52 (1.24–1.85)	< 0.001	1.32 (1.08–1.62)	.007	1.15 (0.92–1.42)	.22
Serum copper, mg/l							
Per unit increase	599/2,503	3.77 (2.44–5.82)	< .001	2.89 (1.83–4.56)	< .001	2.04 (1.22–3.40)	.006
T1 (0.46–1.02)	185/875	Ref		Ref		Ref	
T2 (1.03–1.17)	200/826	1.23 (1.01–1.50)	.04	1.19 (0.97–1.45)	.09	1.13 (0.92–1.38)	.26
T3 (1.18–2.32)	214/802	1.66 (1.36–2.02)	< .001	1.44 (1.18–1.77)	< .001	1.25 (1.00–1.55)	.05
Serum zinc, mg/l							
T1 (0.50–0.89)	248/911	Ref		Ref		Ref	
T2 (0.90–0.98)	159/802	0.62 (0.51–0.76)	< .001	0.67 (0.55–0.82)	< .001	0.68 (0.55–0.83)	< .001
T3 (0.99–1.62)	192/790	0.87 (0.72–1.05)	.15	0.94 (0.77–1.14)	.51	0.96 (0.79–1.16)	.66

CI, confidence interval; HR, hazard ratio; ref, reference; T, tertile

Model 1: Adjusted for age

Model 2: Model 1 plus body mass index, smoking status, history of type 2 diabetes, prevalent coronary heart disease, history of asthma, history of chronic bronchitis, history of tuberculosis, alcohol consumption, socioeconomic status, leisure-time physical activity, total energy intake, intake of fruits, berries and vegetables, and intake of processed and unprocessed red meat

Model 3: Model 2 plus high-sensitivity C-reactive protein

### Association of serum hsCRP with pneumonia

Direct comparisons were made to the association of serum hsCRP with pneumonia risk in the same set of participants. Serum hsCRP was independently associated with pneumonia risk (Supplementary File 2).

### Association of serum Cu/Zn-ratio with pneumonia in subgroups

**Fig. 3:**
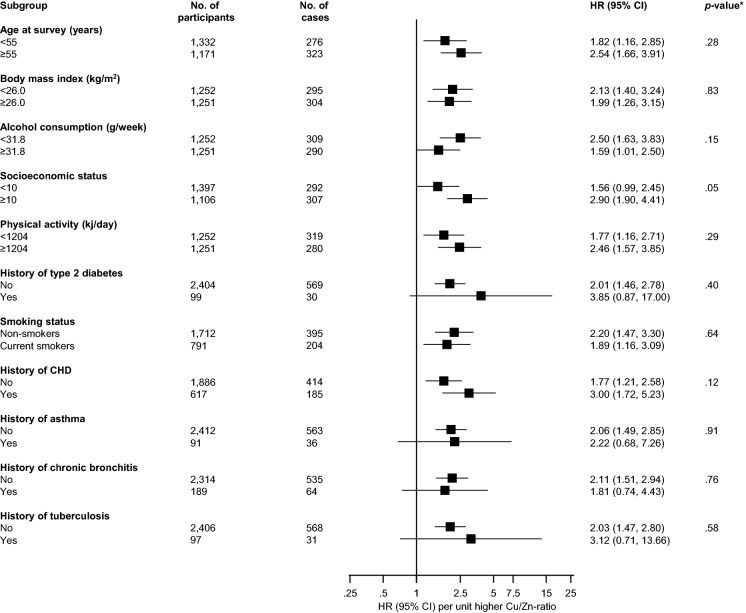
Association between serum Cu/Zn-ratio and pneumonia risk across several clinically relevant subgroups *CHD* coronary heart disease; *CI* confidence interval; *Cu* copper; *HR* hazard ratio; *Zn* zinc

The association between serum Cu/Zn-ratio and pneumonia risk remained consistent across several clinically relevant subgroups except for marginal evidence of interaction by SES (*p* for interaction = 0.05); the association between serum Cu/Zn-ratio and pneumonia risk was strong and positive in men with low SES but was modest in men with high SES (Fig. 3).

### Associations of serum Cu and Zn with pneumonia

A multivariable RCS curve showed that the risk of pneumonia increased linearly with increasing serum Cu across the range 1.60–2.30 (*p*-value for nonlinearity = 0.36) (Fig. 1B). The HR (95% CI) for incident pneumonia per unit increase in serum Cu was 2.89 (1.83–4.56) in analysis adjusted for age, BMI, smoking status, history of type 2 diabetes, prevalent CHD, history of asthma, chronic bronchitis or tuberculosis, alcohol consumption, SES, leisure-time physical activity, total energy intake, intake of fruits, berries and vegetables, and intake of processed and unprocessed red meat, which was attenuated to 2.04 (1.22–3.40) following further adjustment for hsCRP (Table 2). The corresponding adjusted HRs (95% CIs) were 1.44 (1.18–1.77) and 1.25 (1.00–1.55) comparing the top versus bottom tertiles of serum Cu.

A multivariable RCS curve showed a curvilinear relationship between serum Zn and pneumonia risk (*p*-value for nonlinearity = 0.009) (Fig. 1C). Compared to the bottom tertile of Zn, the HRs (95% CIs) for incident pneumonia were 0.67 (0.55–0.82) and 0.94 (0.77–1.14) for the middle and top tertiles of Zn, respectively, in analysis that adjusted for age, BMI, smoking status, history of type 2 diabetes, prevalent CHD, history of asthma, chronic bronchitis or tuberculosis, alcohol consumption, SES, leisure-time physical activity, total energy intake, intake of fruits, berries and vegetables, and intake of processed and unprocessed red meat (Table 2). The respective HRs (95% CIs) were 0.68 (0.55–0.83) and 0.96 (0.79–1.16) in further analysis adjusted for hsCRP. The associations of serum Cu/Zn-ratio, Cu and Zn with risk of pneumonia remained similar in analyses that excluded the first two years of follow-up (Supplementary File 3).

## Discussion

### Key findings

In this prospective evaluation of the relationship between serum Cu/Zn-ratio and risk of incident pneumonia using a cohort of middle-aged and older Finnish men, elevated serum Cu/Zn-ratio was associated with an increased risk of incident pneumonia in a linear dose–response manner. The association did not differ across several clinically relevant subgroups, except for evidence of effect modification by SES; the association appeared to be stronger in men with low SES. In separate evaluations of serum Cu and Zn, Cu was positively associated with pneumonia in a linear dose–response manner, whereas serum Zn was inversely associated with pneumonia risk in a curvilinear manner. When serum Cu/Zn-ratio and Cu were modelled as categorical variables, the associations were attenuated on further adjustment for hsCRP, reflecting the fact that inflammatory pathways are involved in the development of pneumonia and hence confirms the fact that inflammation is a potential mediator of the observed association. Furthermore, correlation analysis demonstrated a strong positive correlation between serum Cu/Zn-ratio and hsCRP. If serum hsCRP is a potential mediator, then adjusting for it constitutes an overadjustment. Findings therefore suggest independent associations of serum Cu/Zn-ratio, Cu and Zn with pneumonia risk. Furthermore, the associations remained persistent when the first two years of follow-up were excluded. In further analysis that assessed the association of serum hsCRP with pneumonia risk in the same set of participants, a relatively weaker association was demonstrated in the analysis that modeled serum hsCRP as a continuous variable; which implies that serum Cu/Zn-ratio may be a stronger risk indicator than serum hsCRP for pneumonia risk.

### Comparison with previous studies

To our knowledge, this is the first study to evaluate the prospective association between serum Cu/Zn-ratio and pneumonia risk, hence, we are unable to compare the current findings in the context of previous work. However, several epidemiological observational studies have demonstrated associations between serum Cu/Zn-ratio and several age-related degenerative conditions such as cardiovascular mortality (Leone et al. 2006; Reunanen and others 1996), HIV-1 mortality (Lai et al. 2001), cancer (Leone et al. 2006), knee chondrocalcinosis (He et al. 2020), and all-cause mortality (Malavolta et al. 2010). In a recent prospective evaluation, Laine and colleagues demonstrated an increased serum Cu/Zn-ratio and Cu concentration to be each associated with an increased risk of incident infections; there was no evidence of an association of Zn with incident infection, except when the analysis was limited to the first 10 years of follow-up (Laine et al. 2020). However, the outcome used in this evaluation comprised a comprehensive list of infectious conditions including intestinal infectious diseases, other bacterial diseases, viral diseases, diseases of the ear, other forms of heart disease, acute respiratory infections, influenza, pneumonia, diseases of the urinary system and male genital organs, and infections of skin and subcutaneous tissue. Though the commonest infection was pneumonia,(Laine et al. 2020) it is uncertain which specific outcome/outcomes could be driving the observed association, as estimates for cause-specific infections were not reported. In a number of case–control studies that were based on patients with bacterial, viral and parasitic infections, serum Cu/Zn-ratio was demonstrated to be a potential prognostic marker (Asemota et al. 2018; Kassu et al. 2006; Van Weyenbergh et al. 2004). Given that this is the first prospective study to evaluate the association between serum Cu/Zn-ratio and pneumonia, other large-scale prospective studies are still needed to confirm the current findings.

### Explanations for findings

Several mechanistic pathways may underline the observed associations of serum Cu/Zn-ratio and Cu and Zn concentrations with the risk of incident pneumonia. In addition to their roles in almost every cellular process in the human body,(Chimienti 2013; Festa and Thiele 2011) Cu and Zn play important roles in the optimal functioning of the immune system. (Stafford et al. 2013) Though Cu plays a beneficial role in numerous biological processes, it can exhibit toxic effects in high amounts. High levels of Cu could increase the risk of infections such as pneumonia via increased inflammation, given its close relationship with ceruloplasmin, which is elevated during an acute phase response,(Uriu-Adams and Keen 2005) in addition to the ability of Cu to serve as a nutrient for infectious microbes. (Besold et al. 2016) For almost six decades, Zn has been known as an important factor for the immune system;(Prasad et al. 1963) its role in immune function has been consistently demonstrated in several cellular studies. (Haase and Rink 2014) The immune defence system relies on two major groups of cells (innate and adaptive immune cells), which also depend on Zn availability at multiple levels. (Wellinghausen et al. 1997) The major roles played by Zn in immunity include (i) signal transduction of immune cells; (ii) its impact on immune cell function such as suppression of several T cell-mediated immune reactions and formation of neutrophil extracellular traps; and (iii) “nutritional immunity”, a host response designed to starve pathogens of essential metals. (Haase and Rink 2014) Consequently, Zn deficiency leads to impaired immune function and an increased risk of infections. With advancing age, there is a decrease in serum Zn concentrations due to insufficient dietary Zn consumption, reduced intestinal absorption or increased losses (due to diarrhoea or use of diuretics) (Mocchegiani et al. 2013) and/or an increase in serum Cu concentrations (Baudry et al. 2020) due to the presence of inflammatory conditions commonly seen in old age. (Sullivan et al. 1979) This consequently leads to an increase in the serum Cu/Zn-ratio. Given that an increased serum Cu/Zn-ratio is commonly seen in older people, there is also a possibility that our findings of an increased risk of pneumonia with an increased serum Cu/Zn-ratio could be due to reverse causation. However, this may be unlikely given that the findings were essentially similar on excluding the first two years of follow-up.

### Implications of findings

The overall evidence suggests that serum Cu/Zn-ratio, Cu and Zn could be risk markers for incident pneumonia. Whether there is a causal relevance to these presented relationships would need to be proved using appropriate study designs such as randomised controlled trials and Mendelian randomisation studies. Nevertheless, the findings are clinically relevant. It has previously been suggested that the serum Cu/Zn-ratio may be a valuable predictive marker for pathological outcomes, and might be comparable or even superior to other well established inflammatory markers such as CRP and erythrocyte sedimentation rate. (Malavolta et al. 2015) Indeed, our analysis showed that serum Cu/Zn-ratio might be a potentially stronger risk indicator for pneumonia risk than hsCRP. An increment of the serum Cu/Zn-ratio above 2.0 in older people has been reported to commonly reflect an inflammatory response or decreased nutritional Zn status. (Malavolta et al. 2010) Measurement of the serum Cu/Zn-ratio as well as serum Cu and Zn concentrations could be used to identify individuals at high risk of serious infections such as pneumonia. However, formal risk prediction analyses are needed to assess the value of these potential risk predictors. Since Zn deficiency in old age is commonly due to insufficient dietary Zn consumption, reduced intestinal absorption or increased losses,(Mocchegiani et al. 2013) its supplementation could help alleviate the deficiencies, which could ultimately provide optimal levels of serum Cu/Zn-ratio in at-risk individuals. There is consistent evidence that preventive Zn supplementation reduces the risk of morbidity and mortality from infectious diseases such as pneumonia, diarrhoea and malaria. (Bates et al. 1993; Yakoob et al. 2011).

### Strengths and limitations

Apart from being the first prospective evaluation of the association between serum Cu/Zn-ratio and the specific outcome of pneumonia, other strengths include (i) the representativeness of the general Finnish middle-aged to older male population, (ii) employment of a relatively large cohort, (iii) the long-term follow-up of the cohort, and (iv) the comprehensive analyses including adjustment for a panel of potential confounders, assessment of the dose–response relationships, evaluation for effect modification on the association using several clinically relevant characteristics and sensitivity analysis. Several limitations of this study deserve consideration. They include (i) the inability to generalise findings to other populations, women and other age groups; furthermore, evidence suggests that there may be gender differences in the concentrations of Cu and Zn;(Olsen et al. 2012) (ii) the possibility that regression dilution bias could have underestimated the associations due to the use of single baseline measurements of the exposures and the long-term follow-up period; (iii) serum Cu concentrations may not accurately reflect actual Cu status, given that leucocyte Cu measurement is considered to be a more reliable index of Cu status in the body;(DiNicolantonio et al. 2018) and (iv) the potential for biases such as residual confounding and reverse causation as with all observational cohort studies.

#### Conclusions

An increased serum Cu/Zn-ratio and serum Cu concentrations are associated with an increased risk of incident pneumonia in middle-aged and older Finnish men, consistent with linear dose–response relationships. The relationship between serum Zn and pneumonia is inverse and curvilinear. Furthermore, serum Cu/Zn-ratio might be a stronger risk indicator for pneumonia than hsCRP, a major inflammatory marker.

## Supplementary Information

Below is the link to the electronic supplementary material.

Supplementary file1 (DOCX 42 KB)

## Data Availability

The data that support the findings of this study are available from the Principal Investigator (J.A.L.) upon reasonable request.

## Abbreviations

BMI: Body mass index
CAP: Community acquired pneumonia
CHD: Coronary heart disease
CI: Confidence interval
COPD: Chronic obstructive pulmonary disease
Cu: Copper
HDL-C: High-density lipoprotein cholesterol
HR: Hazard ratio
hsCRP: High-sensitivity C-reactive protein
IQR: Interquartile range
KIHD: Kuopio Ischemic Heart Disease
SD: Standard deviation
SES: Socioeconomic status
Zn: Zinc
