# Supplementary material for: Serum copper-to-zinc ratio and risk of incident pneumonia in caucasian men: a prospective cohort study
Source: Biometals. 2022 Jul 4;35(5):921–33. doi: 10.1007/s10534-022-00414-4 (PMC9546975; doi:10.1007/s10534-022-00414-4)
Supplement: Supplementary file 1 — Supplementary file1 (DOCX 42 KB) [file 10534_2022_414_MOESM1_ESM.docx]

**Supplementary File 1:** STROBE 2007 Statement—Checklist of items that should be included in reports of cohort studies

| **Section/Topic** | Item # | Recommendation | Reported on page # |
| --- | --- | --- | --- |
| **Title and abstract** | 1 | (*a*) Indicate the study’s design with a commonly used term in the title or the abstract | Page 1 |
|  |  | (*b*) Provide in the abstract an informative and balanced summary of what was done and what was found | Page 2 |
| Introduction | | |  |
| Background/rationale | 2 | Explain the scientific background and rationale for the investigation being reported | Pages 3-4 |
| Objectives | 3 | State specific objectives, including any prespecified hypotheses | Page 4 |
| Methods | | |  |
| Study design | 4 | Present key elements of study design early in the paper | Methods |
| Setting | 5 | Describe the setting, locations, and relevant dates, including periods of recruitment, exposure, follow-up, and data collection | Methods |
| Participants | 6 | (*a*) Give the eligibility criteria, and the sources and methods of selection of participants. Describe methods of follow-up | Methods |
|  |  | (*b*) For matched studies, give matching criteria and number of exposed and unexposed | Not applicable |
| Variables | 7 | Clearly define all outcomes, exposures, predictors, potential confounders, and effect modifiers. Give diagnostic criteria, if applicable | Methods |
| Data sources/ measurement | 8* | For each variable of interest, give sources of data and details of methods of assessment (measurement). Describe comparability of assessment methods if there is more than one group | Methods |
| Bias | 9 | Describe any efforts to address potential sources of bias | Methods |
| Study size | 10 | Explain how the study size was arrived at | Methods |
| Quantitative variables | 11 | Explain how quantitative variables were handled in the analyses. If applicable, describe which groupings were chosen and why | Methods |
| Statistical methods | 12 | (*a*) Describe all statistical methods, including those used to control for confounding | Methods |
|  |  | (*b*) Describe any methods used to examine subgroups and interactions | Methods |
|  |  | (*c*) Explain how missing data were addressed | Not applicable |
|  |  | (*d*) If applicable, explain how loss to follow-up was addressed | Not applicable |
|  |  | (*e*) Describe any sensitivity analyses | Methods |
| Results | | |  |
| Participants | 13* | (a) Report numbers of individuals at each stage of study—eg numbers potentially eligible, examined for eligibility, confirmed eligible, included in the study, completing follow-up, and analysed | Methods |
|  |  | (b) Give reasons for non-participation at each stage | Methods |
|  |  | (c) Consider use of a flow diagram |  |
| Descriptive data | 14* | (a) Give characteristics of study participants (eg demographic, clinical, social) and information on exposures and potential confounders | Results; Table 1 |
|  |  | (b) Indicate number of participants with missing data for each variable of interest |  |
|  |  | (c) Summarise follow-up time (eg, average and total amount) | Results |
| Outcome data | 15* | Report numbers of outcome events or summary measures over time | Results |
| Main results | 16 | (*a*) Give unadjusted estimates and, if applicable, confounder-adjusted estimates and their precision (eg, 95% confidence interval). Make clear which confounders were adjusted for and why they were included | Results; Table 2; |
|  |  | (*b*) Report category boundaries when continuous variables were categorized | Results; Table 2 |
|  |  | (*c*) If relevant, consider translating estimates of relative risk into absolute risk for a meaningful time period |  |
| Other analyses | 17 | Report other analyses done—eg analyses of subgroups and interactions, and sensitivity analyses | Results; Figure 3 |
| Discussion |  |  |  |
| Key results | 18 | Summarise key results with reference to study objectives | Discussion |
| **Limitations** |  |  |  |
| Interpretation | 20 | Give a cautious overall interpretation of results considering objectives, limitations, multiplicity of analyses, results from similar studies, and other relevant evidence | Discussion |
| Generalisability | 21 | Discuss the generalisability (external validity) of the study results | Discussion |
| Other information |  |  |  |
| Funding | 22 | Give the source of funding and the role of the funders for the present study and, if applicable, for the original study on which the present article is based | After Discussion |

**Supplementary File 2.** Association of hsCRP with risk of pneumonia

| **Exposure** | **Events/**  **Total** | **Model 1** |  | **Model 2** |  | **Model 3** |  |
| --- | --- | --- | --- | --- | --- | --- | --- |
|  |  | HR (95% CI) | *P-*value | HR (95% CI) | *P-*value | HR (95% CI) | *P-*value |
| Per unit increase in log hsCRP | 599 / 2,503 | 1.37 (1.26-1.49) | < 0.001 | 1.24 (1.13-1.36) | < .001 | 1.17 (1.06-1.29) | .002 |
| T1 (0.10-0.87) | 167 / 851 | ref |  | ref |  | ref |  |
| T2 (0.88-1.95) | 215 / 824 | 1.55 (1.27-1.90) | <.001 | 1.44 (1.17-1.78) | .001 | 1.39 (1.13-1.72) | .002 |
| T3 (>1.95) | 217 / 828 | 1.93 (1.58-2.37) | < .001 | 1.59 (1.28-1.97) | <.001 | 1.40 (1.11-1.76) | .004 |

CI, confidence interval; HR, hazard ratio; hsCRP, high sensitivity C-reactive protein; ref, reference; T, tertile

Model 1: Adjusted for age

Model 2: Model 1 plus body mass index, smoking status, history of type 2 diabetes, prevalent coronary heart disease, history of asthma, history of chronic bronchitis, history of tuberculosis, alcohol consumption, socioeconomic status, leisure-time physical activity, total energy intake, intake of fruits, berries and vegetables, and intake of processed and unprocessed red meat

Model 3: Model 2 plus serum copper-to-zinc ratio

**Supplementary File 3.** Associations of serum copper, zinc and copper-to-zinc ratio with risk of pneumonia, on exclusion of the first two years of follow-up

| **Exposure** | **Events/**  **Total** | **Model 1** |  | **Model 2** |  | **Model 3** |  |
| --- | --- | --- | --- | --- | --- | --- | --- |
|  |  | HR (95% CI) | *P-*value | HR (95% CI) | *P-*value | HR (95% CI) | *P-*value |
| **Serum copper-to-zinc ratio** | | | | | | | |
| Per unit increase | 588 / 2,454 | 2.51 (1.85-3.41) | < 0.001 | 1.98 (1.44-2.73) | < .001 | 1.56 (1.10-2.22) | .012 |
| T1 (0.48-1.07) | 175 / 818 | ref |  | ref |  | ref |  |
| T2 (1.08-1.27) | 198 / 820 | 1.15 (0.93-1.40) | .19 | 1.09 (0.89-1.34) | .42 | 1.03 (0.84-1.26) | .79 |
| T3 (1.28-3.12) | 215 / 816 | 1.48 (1.21-1.81) | < 0.001 | 1.29 (1.05-1.58) | .02 | 1.11 (0.89-1.38) | .35 |
| **Serum copper, mg/l** | | | | | | | |
| Per unit increase | 588 / 2,454 | 3.55 (2.28-5.52) | < .001 | 2.68 (1.68-4.26) | < .001 | 1.85 (1.10-3.11) | .02 |
| T1 (0.46-1.02) | 184 / 865 | ref |  | ref |  | ref |  |
| T2 (1.03-1.17) | 195 / 808 | 1.21 (0.99-1.48) | .07 | 1.17 (0.95-1.43) | .14 | 1.10 (0.90-1.35) | .36 |
| T3 (1.18-2.32) | 209 / 781 | 1.64 (1.35-2.00) | < .001 | 1.42 (1.16-1.74) | .001 | 1.22 (0.98-1.53) | .07 |
| **Serum zinc, mg/l** | | | | | | | |
| T1 (0.50-0.89) | 243 / 891 | ref |  | ref |  | ref |  |
| T2 (0.90-0.98) | 156 / 785 | 0.62 (0.51-0.76) | <.001 | 0.67 (0.55-0.82) | <.001 | 0.68 (0.55-0.83) | <.001 |
| T3 (0.99-1.62) | 189 / 778 | 0.87 (0.72-1.05) | .16 | 0.94 (0.78-1.15) | .56 | 0.96 (0.79-1.17) | .72 |

CI, confidence interval; HR, hazard ratio; ref, reference; T, tertile

Model 1: Adjusted for age

Model 2: Model 1 plus body mass index, smoking status, history of type 2 diabetes, prevalent coronary heart disease, history of asthma, history of chronic bronchitis, history of tuberculosis, alcohol consumption, socioeconomic status, leisure-time physical activity, total energy intake, intake of fruits, berries and vegetables, and intake of processed and unprocessed red meat

Model 3: Model 2 plus high-sensitivity C-reactive protein
